# Influence of solids and hydraulic retention times on microbial diversity and removal of estrogens and nonylphenols in a pilot-scale activated sludge plant

**DOI:** 10.1016/j.heliyon.2023.e19461

**Published:** 2023-08-28

**Authors:** Lawson Mensah, Bruce Petrie, Mark Scrimshaw, Elise Cartmell, Mandy Fletton, Pablo Campo

**Affiliations:** a Environmental Science Department, Kwame Nkrumah University of Science and Technology, Kumasi, Ghana; b Robert Gordon University, Garthdee Rd, Garthdee, Aberdeen, AB10 7AQ, UK; c Department of Life Sciences, Brunel University London, Uxbridge, UB8 3PH, UK; d Scottish Water, Castle House, 6 Castle Drive, Carnegie Campus, Dunfermline, KY11 8GG, UK; f UKWIR Limited, 50 Broadway, London, SW1H 0RG, UK; e Cranfield Water Science Institute, School of Water, Energy & Environment, Cranfield University, MK43 0AL, UK

**Keywords:** Microbial diversity, Phospholipids fatty acids, 16S rRNA analysis, Estrogens, Nonylphenols, Activated sludge plant

## Abstract

The removal of EDCs in activated sludge processes can be enhanced by increasing solid and hydraulic retention times (SRT and HRT); it has been suggested that the improvement in removal is due to changes in microbial community structure (MCS). Though the influence of SRT and HRT on chemical removal and MCS has been studied in isolation, their synergistic impact on MCS and the removal of estrogens and nonylphenols in activated sludge remains unknown. Hence, we investigated how both parameters influence MCS in activated sludge processes and their ulterior effect on EDC removal. In our study, an activated sludge pilot-plant was fed with domestic sewage fortified with 100 and 1000 ng/L nonylphenols or 2 and 15 ng/L estrogens and operated at 3, 10 and 27 d SRT (constant HRT) and at 8, 16 and 24 h HRT (constant SRT). The MCS was assessed by phospholipid fatty acids (PLFA) analysis, and the archaeal and bacterial diversities were determined by 16S rRNA analysis. From the PLFA, the microbial abundance ranked as follows: Gram-negative > fungi > Gram-positive > actinomycetes whilst 16S rRNA analysis revealed *Proteobacteria* > *Bacteroidetes* > Others. Both PLFA and 16S rRNA analysis detected changes in MCS as SRT and HRT were increased. An SRT increment from 3 to 10 d resulted in higher estrone (E1) removal from 19 to 93% and nonylphenol-4-exthoxylate (NP_4_EO) from 44 to 73%. These findings demonstrate that EDC-removal in activated sludge plants can be optimised where longer SRT (>10 d) and HRT (>8 h) are suitable. We have also demonstrated that PLFA can be used for routine monitoring of changes in MCS in activated sludge plants.

## 1 Introduction

The discharge of endocrine disrupting chemicals (EDCs) such as estrogens and nonylphenols from wastewater treatment plants (WWTPs) into surface waters is of great environmental concern. EDCs can induce feminine characteristics in male aquatic organisms [1,2], accumulate in the environment [3], and their presence in effluent will continue to impede the interest in indirect reuse of reclaimed municipal effluent [[4], [5], [6]]. Research in this field shows that the removal of EDCs by WWTPs can be optimised by changing operational parameters such as solids retention time (SRT) [7,8], hydraulic retention time (HRT) [8], temperature, dissolved oxygen and process layout [9,10]. In activated sludge plants (ASP), SRT increase resulted in higher removal of estrogens [[11], [12], [13]] and nonylphenols (ethoxylates and carboxylates) [13]; HRT increases also resulted in better removal of alkylphenols and estrogens [14,15].

The improved removals at higher SRT and HRT have been attributed to increases in microbial activities, sorption to sludge or other chemical removal mechanisms that hinge on higher biomass concentrations. It has been suggested that longer SRTs facilitate slow-growing microbes which may led to a more diverse microbial community where EDC degraders can thrive [[16], [17], [18]]. An increase in HRT leads to improved removal because of a longer contact time between substrates and microorganisms and reducing the chemical loading rate, which causes the microorganisms to compete and work harder for the limited resources available [15,19]. The effects of SRT and HRT on microbial population and diversity in WWTPs have also been studied but often in isolation. For example, reducing HRT from 30 to 5 h resulted in 20% decrease in ammonia-oxidising-bacteria and 11% increase in nitrite-oxidising-bacteria [20]. Furthermore, *Betaproteobacteria* and *Bacteroidetes* were dominant at low SRTs, whereas *Delta* and *Epsilon proteobacteria* were dominant at higher SRTs [21,22].

Microbial community structural studies in wastewater treatment systems are often carried out with methods such as 16S rRNA analysis [23] for detailed identification of archaeal and bacterial genera or denaturing gradient gel electrophoresis (DGGE) and Biolog [24] analysis for broad characterisation of microbial communities; the use of phospholipid fatty acids (PLFA) in WWTP samples is rare. PLFA has been used for assessing microbial community structure in soil samples for over three decades [25] and continue to be used because of its effectiveness in detecting changes in microbial communities due to stress or an intervention [26] and has been successfully applied to WWTP samples from different geographical and temporal regions [27] and in a study of different process designs [28]. In PLFA analysis, the abundance of fatty acids extracted from microbial cell membranes is used as an indicator of Gram-positive, Gram-negative, actinobacteria and fungi abundance [29]. Although interpretation of the results has known limitations [30], it remains widely applied and accepted for characterising changes in microbial communities [31].

Missing from the literature is the combined effect of SRT and HRT on microbial community structure and removal of EDCs in activated sludge processes. To address this knowledge gap, we conducted pilot trials to replicate varying operational parameters under conditions such that the response of the microbial communities and the corresponding EDC removals could be confirmed. Our research sought to prove that both longer SRT and HRT can improve MCS and thus enhance the removal of estrogens and nonylphenols. Temperature is known to influence microbial diversity and chemical removal in activated sludge plants, and it was not controlled in this study. However, the pilot set-up was housed in a temperature-controlled laboratory, hence the temperature did not vary widely.

## 2 Materials and methods

### 2.1 Pilot-plant configuration, operation and sampling

**Fig. 1:**
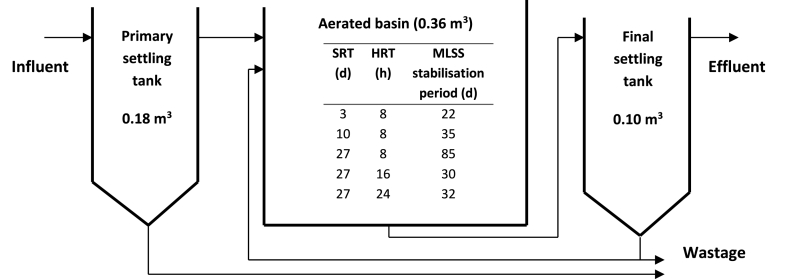
Pilot-scale activated sludge plant schematic and experimental design for SRT and HRT changes.

A pilot-scale ASP consisting of a primary sedimentation tank, an aerated basin and a final clarifier (Fig. 1) was fed with domestic crude sewage from a nearby WWTP with a population equivalent of 3000. The initial HRT was kept at 8 h whilst SRTs of 3, 10 and 27 d, typical of full-scale activated sludge plants [13] were studied. The SRT was controlled through wastage of return activated sludge (RAS), the recycle ratio of RAS to the settled sewage flow rate was 0.60, and the mixed liquor suspended solids (MLSS) and RAS were measured daily to assess the sludge age. For each SRT experiment, the activated sludge reactor was allowed a stabilisation period of at least three sludge ages. The HRT studies were also conducted at 8, 16 and 24 h to include the typical full-scale range whilst SRT was maintained at 27 d. The stabilisation period was at least one sludge age [13].

One litre sample was collected in triplicate each day from the settled sewage and final effluent for seven consecutive days for biochemical oxygen demand (BOD), chemical oxygen demand (COD), total suspended solids (TSS), ammonia (NH_3_), estrogens and nonylphenols analyses. Mixed liquor sample (2.5 L) was also collected for PLFA and 16S rRNA analyses.

### 2.2 Methods for the analysis of sanitary determinants

Chemical oxygen demand (COD), ammonia (NH_4_^+^-N) and nitrate (NO_3_^−^-N) were measured with cell test kits from VWR International (Leicestershire, UK) and subsequently detected by spectrophotometry. Suspended and volatile solids as well as biochemical oxygen demand (BOD) were determined following Standard Methods [32].

### 2.3 Method for the analysis of phospholipid fatty acids analysis

#### 2.3.1 Chemicals

Toluene:methanol (1:1), 0.56 g KOH in 50 mL CH_3_OH, 59 mL/L acetic acid; Hexane:chloroform (4:1); citrate buffer was prepared by dissolving 7.2 g of anhydrous citric acid and 11 g tri-sodium citrate in 250 mL ultrapure deionised water (18.2 MΩ quality, Elga, Marlow, UK). Bligh-and-Dyer solvent (21% citrate buffer, 26% chloroform, 53% methanol v/v/v) containing 30 mg of butylhydroxytoluene; 99.9% purity chloroform, toluene, ammonium hydroxide and ammonium acetate (Sigma Aldrich, Dorset, UK).

#### 2.3.2 Sample preparation and analysis

Around 250 mL of MLSS was transferred into Nalgene bottle and centrifuged at 8000 rpm for 20 min at 4 °C. Approximately eighty percent of the supernatant was decanted, and the sludge was re-suspended in the remaining supernatant and transferred into a 50-mL plastic sample bag, kept at −80 °C for 48 h followed by freeze-drying at −50 °C and then stored until analysis. The PLFA analysis followed the method described by Ref. [25]. The phospholipid in 0.5 g of freeze-dried sludge were extracted with Bligh-and-Dyer solvent, fractionated by solid-phase extraction and converted to fatty acid methyl esters (FAMEs) by mild alkaline methanolysis. The FAMEs were then analyzed by GC–FID and quantified.

### 2.4 16S rRNA analysis

The 16S rRNA analysis and the subsequent bioinformatic processing of the data were carried out by PROKARYA LTD, Newcastle upon Tyne, UK (Company Number: 09612606, Dissolved in 2019). In their method, 250 μL of sludge sample was buffered and ribolysed. The DNA suspension was precipitated, bound, washed and air dried before re-suspending in 50 μL DES to elute and to solubilise the DNA. 0.5 μL of DNA extract was added to a PCR mix containing reaction buffer, enzyme blend, dNTPs, reverse primer 926r (5′-CCGGYCAATTYYMMTTTTRAGTTT-3′), a unique a 12-base pair Golay bar-coded forward-primer 515F (5′-GTGNCAGMGCCGCGGTAA-3′) including a sequencing adaptor and a GT spacer which was followed by a polymerase chain reaction. The PCR product was purified and pooled to form a single PCR library containing equimolar amounts prior to sequencing in the Ion Torrent Personal Genome Machine. The raw data of microbial abundance was provided to the researcher for statistical analysis.

### 2.5 Methods for the analysis of steroid estrogens and nonylphenols

#### 2.5.1 Chemicals

Analytes of interest were estrone (E1), estradiol (E2), estriol (E3), estrone-1,3-sulfate (E1-3S) and ethynylestradiol (EE2) as well as nonylphenols (including ethoxylates and carboxylates). Analytical grade (99.9% purity) estrone (E1), 17β-estradiol (E2), estriol (E3), 17α-ethynylestradiol (EE2), estrone sulfate (E1-3S) from Sigma Aldrich (Dorset, UK). 99.9% pure hexane, dichloromethane (DCM), ethyl acetate (EtOAc), and methanol (MeOH) from Rathburn Chemicals (Walkerburn, UK). Two ng/L mixed estrogens, 15 ng/L mixed estrogens, EtOAc:hexane (10:90), 3% NH_4_OH in MeOH, DCM:MeOH (90:10), ultrapure (UP) water:MeOH (80:20) containing 0.1% NH_4_OH and 0.1% acetic acid in MeOH. Deuterated internal standards estrone-2,4,16,16-d_4_, 17β-estradiol-2,4,16,16,17-d_5_, estriol-2,4,17-d_3_, 17α-ethinylestradiol-2,4,16,16-d_4_, sodium estrone-2,4,16,16-d_4_ sulfate (QMX Laboratories, Thaxted, UK). Technical 4-NP, 4-nonylphenol-mono-ethoxylate, nonylphenol di-ethoxylate, long chain NPEOs mixtures CO210, CO520 and CO720 (Sigma Aldrich, Dorset, UK), nonylphenoxy acetic acid (QMX Laboratories, Thaxted, UK), HPLC grade (99.9% purity) acetone, dichloromethane (DCM), ethyl acetate (EtOAc), hexane, methanol (MeOH) and acetonitrile (ACN) (Rathburn Chemicals, Walkerburn, UK). Nonylphenol spike concentrations were 100 and 1000 ng/L of mixed compounds.

#### 2.5.2 EDCs analysis

Both estrogens and nonylphenols were analyzed as described elsewhere [33,34]. Briefly, for estrogens 1-L samples of settled final effluent were filtered through 1.2 μm GF/C filter paper (VWR, Lutterworth, UK); for nonylphenols, samples (250 mL of final effluent or 100 mL of settled sewage) were filtered through 1.2 μm GF/C filter paper (VWR, Lutterworth, UK). In both cases, filtrates were put through solid phase extraction cartridges and detection and quantification of target analytes were done by UPLC-MS/MS (Waters Acquity UPLC, Waters, Manchester, UK). The instrument was operated under multiple reaction monitoring (MRM) mode; fragmentation reactions and instrumental parameters are included in the supporting information.

### 2.6 Statistical analysis of the data

The proportion of Gram-positive (GRAM+) bacteria in each sample was quantified as the sum of relative abundances of the following methyl esters of fatty acids: 14:0i, 15:0i, 15:0ai, 16:0i, 17:0i and 17:0ai; Gram-negative (GRAM-) bacteria by 16:1ω7c, 15:1ω4, 16:1ω9c, cy19:0, cy17:0, 17:1ω9c, and 18:1ω9c, 18:1ω7t; actinomycetes (ACTS) by 10Me16:0, 10Me17:0 and 10Me18:0; and fungi abundance by 16:1ω5c, 18:1ω9c, 18:2ω6, 18:3ω3 and 20:5ω3 [35]. The Simpson index of microbial diversity (expressed as α-diversity) measures the richness and evenness of species in a system. It ranks from zero to one, with zero being infinite diversity and one representing no diversity. The α-diversity in the pilot-scale reactor under each experimental condition was calculated in Microsoft Excel (2016) as follows [36]: $Simpson\;index\;\left(D\right)=\frac{\sum_{i=1}^{R}n_{i}(n_{i}-1)}{N\left(N-1\right)}$.

(1) $$Simpson\;index\;\left(D\right)=\frac{\sum_{i=1}^{R}n_{i}(n_{i}-1)}{N\left(N-1\right)}$$

n_i = numberofindividualsinonetypeofspecies

N = total number of individuals in a system.

Principal component analysis (PCA) was used to assess multivariate correlations between the microbial diversity, mean abundance, and removal of the steroid estrogens and nonylphenols under each experimental set-up using Statistica 13 (TIBCO Software).

## 3 Results and discussion

### 3.1 Pilot-plant performance at different SRTs and HRTs

For studies undertaken at constant HRT (8) the pilot-plant achieved removals of 74% BOD, 62% COD, 6% ammonia and 37% suspended solids; no nitrification at 3 d SRT was observed. At 10 d SRT, the reactor was nitrifying, with final effluent ammonia concentrations <1 mg/L and percentage removals increased significantly (*p* < 0.001, n = 7) to 88% BOD, 82% COD, 99% ammonia and 76% suspended solids. These removals were significantly different (*t*-test) under each of the SRTs studied except for the removal of COD (*p* > 0.05, n = 7) and ammonia (*p* > 0.05, n = 7) between 10 and 27 d SRT. The low removal of BOD, COD, SS and ammonia at 3 d SRT and 8 h HRT, is due to a high influent chemical loading rate and low biomass available for the degradation and sorption of the bulk organic matter. These conditions also led to a high F:M ratio causing most of the nutrients to pass through the plant without being degraded.

For studies where the HRT was varied at constant SRT (27 d), 94–96% of BOD and 82–84% of COD removals were observed, and all simulations achieved nitrification (Table 1). There was insignificant variation in the removals of ammonia or COD at each HRT studied, and no significant difference in BOD (*p* > 0.05, n = 7) at 16 and 24 h HRTs. The performance of the pilot-plant is comparable to observations by Petrie et al. [13] in a similar study.

**Table 1:** Performance of the pilot-plant in each experimental set-up.

Pilot plant set-up		BOD_5_			COD			Ammonia			Suspended solids			Nitrates	
		Settled (mg/L)	Final effluent (mg/L)	Mean removal (%)	Settled (mg/L)	Final effluent (mg/L)	Mean removal (%)	Settled (mg/L)	Final effluent (mg/L)	Mean removal (%)	Settled (mg/L)	Final effluent(mg/L)	Mean removal (%)	Settled (mg/L)	Final effluent (mg/L)
**SRTstudies** **@ 8h HRT**	**3 d**	127 ± 20	32.7 ± 6.1	74.3 ± 2.4	458 ± 28	172 ± 13	62.4 ± 3.1	51.5 ± 2.9	48.4 ± 2.4	5.9 ± 1.0	140 ± 9	88.3 ± 4.7	37.0 ± 4.1	0.75 ±0.18	0.79 ±0.09
	**10 d**	120 ± 7.3	14.6 ± 1.6	87.8 ± 2.4	510 ± 83	91.1 ± 13	81.9 ± 3.6	50.8 ± 2.8	0.3 ± 0.1	99.5 ± 0.2	127 ± 5	30.1 ± 5.3	76.3 ± 4.1	1.10 ±0.52	4.55 ±0.56
	**27 d**	123 ± 14	4.8 ± 1.3	96.1 ± 0.7	442 ± 46	74.9 ± 9.6	82.9 ± 2.4	50.9 ± 1.7	0.2 ± 0.1	99.5 ± 0.1	142 ± 7	18.0 ± 4.2	87.4 ± 2.6	0.80 ±0.45	15.2 ±0.85
**HRT studies** **@ 27 d SRT**	**8 h**	123 ± 14	4.8 ± 1.3	96.1 ± 0.7	442 ± 46	74.9 ± 10	82.9 ± 2.4	50.9 ± 1.7	0.2 ± 0.07	99.5 ± 0.14	142 ± 7	18.0 ± 4.2	87.4 ± 2.6	0.80 ±0.45	15.2 ±0.85
	**16 h**	103 ± 9	6.1 ± 2.1	94.1 ± 1.8	363 ± 19	56.4 ± 3.9	84.4 ± 1.0	37.0 ± 3.7	0.2 ± 0.03	99.6 ± 0.08	109 ± 21	27.9 ± 4.8	74.4 ± 3.1	0.55 ±0.4	25.3 ±1.2
	**24 h**	102 ± 31	4.1 ± 2.0	95.6 ± 2.8	367 ± 25	65.5 ± 8.9	82.2 ± 1.4	35.6 ± 5.5	0.1 ± 0.03	99.6 ± 0.09	89.0 ± 7	29.6 ± 4.5	67.0 ± 3.5	0.4 ±0.25	25.4 ±1.7

BOD_5_ = Biochemical oxygen demand with 5-day incubation period; COD = Chemical oxygen demand.

### 3.2 Microbial community structure at constant HRT and varying SRT from PLFA analysis

Under all experimental conditions, the order of microbial abundance ranked as Gram-negative **>** fungi **>** Gram-positive **>** actinomycetes; a similar trend reported by Amir et al. [37] in sludge cake samples. The high abundance of Gram-negative bacteria is probably due to their naturally high proportion in environmental samples. Increasing SRT from 3 to 10–27 d coincided with significant (*p* < 0.01, n = 5) changes in fatty acid profile and the microbial community structure in the pilot plant (Supplementary Figure1). An increase in SRT from 3 to 10 d coincided with a decrease in Gram-positive and Gram-negative bacteria abundance by 5 and 6% respectively, whereas fungi abundance increased by 10.7% along with a significant (*p* < 0.01, n = 7) change in the abundance of actinomycetes from 0.15 to 0.52%. Further elongation of SRT to 27 d had no effect (*p* > 0.05, n = 7) on Gram-positive bacterial abundance; significant reductions in actinomycetes (69%) and fungal (8.7%) and slight increase in Gram-negative bacteria (7.8%) were observed.

**Fig. 2:**
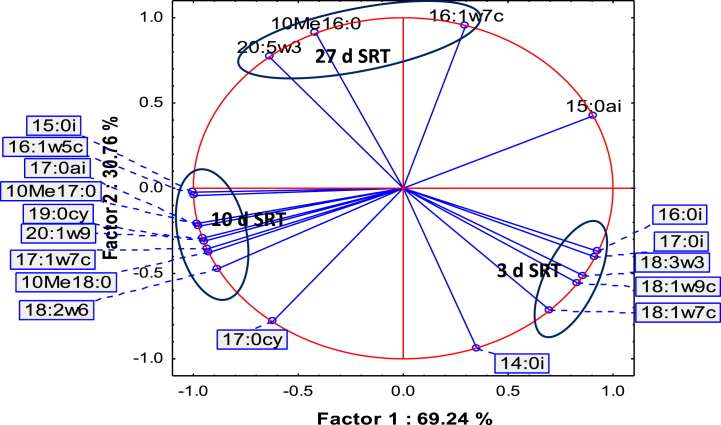
PCA of microbial diversity from fatty acids profiles at constant HRT and 3, 10 and 27 d SRT (n = 7).

Fatty acids cy17:0 and 16:1ω7c correlated (R^2^ = 0.86940) as SRT increased from 3 to 27 d. The cy17:0 is a modification of 16:1ω7c and is formed due to starvation in the reactor [38]. This correlation indicates that increasing the SRT reduces the food-to-microorganisms ratio and causes microbial diversity to change. PCA of the microbial diversities emanating from the fatty acids as variables (Fig. 2) demonstrates that the microbial communities were dissimilar at 3, 10 and 27 d SRT. The community at 27 d had similar fungal profile to that at 3 d and similar Gram-positive profile to that at 10 d SRT. At 3 d SRT, the fatty acids 16:0i, 17:0i, 18:3ω3, 18:1ω9c and 18:1ω7c had high abundances. At 10 d SRT, the dominant fatty acids were 15:0i, 16:1ω5c, 17:0ai, 10Me17:0, 19:0cy, 20:1ω9, 17:1ω7c, 10Me18:0 and 18:2ω6. The 27 d SRT investigation had high percentages of 16:1ω7c, 10Me16:0 and 20:5ω3 fatty acids.

### 3.3 Microbial community structure at constant SRT and varying HRT from PLFA analysis

At a constant SRT, HRT changes also caused shifts in microbial diversity (Supplementary Figure 2). As HRT was raised from 8 to 16 h the fungi proportion increased by 5.1% and Gram-negative bacteria reduced by of 6.0%. Further elongation of HRT to 24 h reduced the proportion of fungi and Gram-positive bacteria by 2.8% and 1.6% respectively, with a corresponding increase in Gram-negative bacteria of1.4%. These changes in microbial groups were insignificant although the fatty acid variations were significant (*p* < 0.002). The order of fatty acids abundances was as that found in the varying SRT studies, except for 18:3ω3 which became the second highest biomarker by increasing from 1.2% at 16 h to 6.9% at 24 h HRT. These changes in fatty acid abundances suggest that HRT increases caused shifts in microbial diversity.

**Fig. 3:**
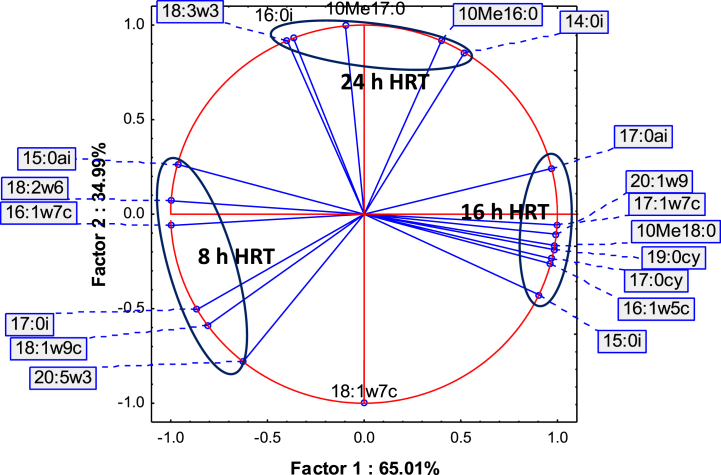
PCA of microbial diversity from PLFA at 8, 16 and 24 h HRT (n = 7).

The outcome of the PCA (Fig. 3) of fatty acids at 8, 16 and 24 h, suggests that the microbial communities were different. At 8 h HRT, fatty acids 15:0ai, 17:0i, 16:1ω7c, 18:2ω6, 18:1ω9c and 20:5ω3 dominated in the microbial communities, while fatty acids 15:0i, 16:1ω5c, 17:0ai, 17:0cy, 19:0cy, 10Me18:0, 17:1ω7c and 20:1ω9 were most abundant at 16 h HRT. At 24 h HRT, fatty acids 16:0i, 10Me16:0, 10Me17:0 and 18:3ω3 were higher in abundance.

In summary, SRT significantly changed the fatty acid profile and the resulting microbial diversity in the pilot-scale reactor; confirming the suggestion that longer SRT allows the slow-growing bacteria to increase in abundance [16]. HRT increases also significantly influenced the fatty acid profile and resulted in moderate changes in the microbial diversity. Although this observation does not affirm the proposition that shorter retention and its consequential less contact time favour fast-growing microbes. One can argue that the PLFA method was not sensitive enough to detect overall microbial changes as HRT increases occurred. Also, studying HRT changes at such a high constant SRT of 27 d resulted unhelpfully in finding the real influence of HRT. The implication is that when operating an activated sludge plant at low HRT, SRT variations are critical for microbial diversity. At high SRT, however, HRT changes produces negligible changes in microbial diversity. Therefore, WWTPs with enough settling capacity should operate at high SRT (>10 d) to ensure greater microbial diversity in the reactor as HRT is more difficult to control due to the changes in influent flow rate.

### 3.4 Simpson's indices (α-diversity) at varying SRT and HRT from 16S rRNA analysis

**Fig. 4:**
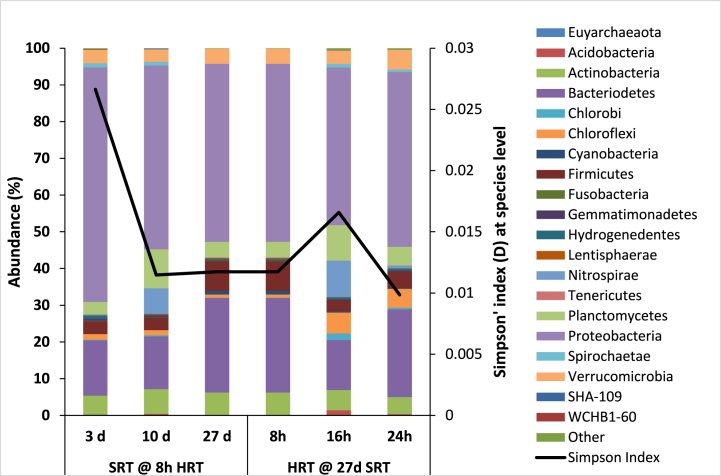
Bacterial and archaeal diversity at phylum level at 3,10 and 27 d SRT (at a constant 8 h HRT) (n = 1); and at 8, 16 and 24 h HRT (at a constant 27 d SRT) (n = 1) with Simpson diversity indices at species level (Equation (1)).

As SRT and HRT increased, the α-diversity of bacterial and archaeal species generally improved which reduced the Simpson diversity index (Equation (1)) accordingly in Fig. 4 [17]. Operating the pilot-plant at 27 d SRT and 24 h HRT produced microbial community which is 2.7 times more diverse than that produced at 3 d SRT and 8 h HRT. One limitation of this study was that the samples analyzed under each condition were not replicated, hence the significance of the variation could not be determined.

### 3.5 Bacterial and archaeal diversity from 16S rRNA analysis at different SRT and HRT

#### 3.5.1 Bacterial diversity at the phylum level

The bacterial diversity in the pilot-scale ASP reactor was dominated by *Proteobacteria* followed by *Bacteroidetes* (Fig. 4) under all conditions, as found by others [39] but the third most abundant phylum was different in each trial. At 3 d SRT, the reactor contained 63% *Proteobacteria*, this abundance was reduced to 50% when SRT was extended to 10 d and decreased slightly to 48% when SRT was furthered to 27 d. The reduction in *Proteobacteria* abundance corresponded with increases in *Nitrospirae* and *Planctomycetes* at 10 d, and *Firmicutes* at 27 d SRT. Fig. 4 also showed that *Bacteroidetes* changed from 26% at 8 h to 14% at 16 h and then back to 24% at 24 h HRT. *Nitrospirae* and *Planctomycetes* increased from 0 to 4% to approximately 10% each as HRT increased from 8 to 16 h. These significant increases in *Nitrospira* and *Planctomycetes* abundance could have caused a reduction the bacterial diversity in the activated sludge reactor, leading to upward and downward trend in the Simpson's indices (Equation (1)) in Fig. 4. The *Proteobacteria* decreased further to 42% at 16 h but recovered back to 48% at 24 h HRT.

**Fig. 5:**
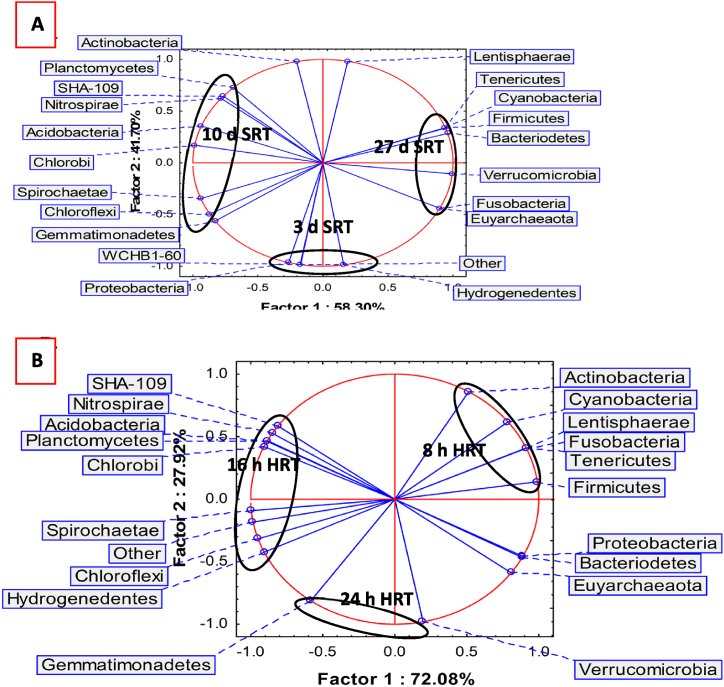
[A] PCA for the comparison of α-diversity in the bacteria communities at 3, 10 and 27 d SRT; and [B] PCA of bacterial and archaeal phylum diversity at 8, 16 and 24 h HRT..

The PCA output (Fig. 5, panel A) indicates that the bacterial diversity at each SRT was distinct, and that *Proteobacteria*, *Hydrogenedentes* and WCHB1-60 had their highest abundance at 3 d SRT. When operating at constant SRT, the variation in HRT also induced changes in the bacterial diversity. Fig. 5, panel B shows that α-diversity at 8, 16 and 24 h HRT are different. The bacterial phyla distribution illustrates that at the diversity at 8 and 24 h HRT had similar abundances of *Bacteroidetes*, *Proteobacteria*, and *Euyarchaeaota* while that of 16 h shared similar abundances of *Chloroflexi*, *Hydrogenedentes* and *Spirochaetae* to the 24 h HRT.

#### 3.5.2 Bacterial diversity at the class and species levels

At constant HRT of 8 h, the dominant species were *Thiothrix eikelboomii*, *Nitrospira* sp. and an uncultured *Comamonadaceae* species at 3, 10 and 27 d SRT, respectively. From 3 to 10 d SRT, *Nitrospirae* abundance increased from 0.2 to 6.94%, and *Thiothrix eikelboomii* abundance reduced from 11.9 to 1.70% at 10 d and to 0.66% at 27 d SRT. With *Proteobacteria* accounting for 42–63% of bacterial abundance in the reactor, the diversity within *Proteobacteria* was of interest. At 3 d SRT, *Alpha, Beta* and *Gammaproteobacteria* were 20, 16 and 23% respectively and the other most dominant genera were uncultured *Comamonadaceae*, *Flavobacterium*, uncultured *Chitiniphagaceae* and uncultured *Rhodobacteraceae*. At 10 d SRT *Proteobacteria* abundance dropped to 50%, and the Alpha, Beta and *Gammaproteobacteria* abundances were 22, 12 and 7%. This increase in SRT had minimal affect the presence of *Alphaproteobacteria*, slightly decreased the *Betaproteobacteria* abundance and decimated the *Gammaproteobacteria* population. Other dominant genera were uncultured *Comamonadaceae*, *Planctomyces*, *Rhodobacter* and *Haliscomenobacter*. At 27 d SRT, *Flavobacterium*, uncultured *Rhodobacteraceae*, *Hydrogenophaga* and *Leadbetterella* were prominent. Overall, *Planctomycetes* abundance fluctuated, but *Firmicutes* increased with increasing SRT.

At constant SRT (of 27 d) the increases in HRT caused the dominant species to change from the species from *Comamonadaceae* family at 8 h to *Nitrospira* sp. at 16 h and then to the uncultured *Comamonadaceae* species. Variation within *Proteobacteria* indicates that HRT increase had no effect on the order of dominance but affected the percentage abundances. At 8 and 24 h HRT, the dominant *order* was *Burkholderiales* with approximately 14 and 13% abundances. *Thiothrichales* also increased from 0.78% at 8 h to 4% at 16 and 24 h HRT. Changes at species level, with the dominance of the *Thiothrix eikelboomii* at 3d SRT which diminishes at 10 d SRT as *Nitrospira* sp. becomes dominant (Supplementary Figure 3).

### 3.6 Removal of estrogens and nonylphenols

**Fig. 6:**
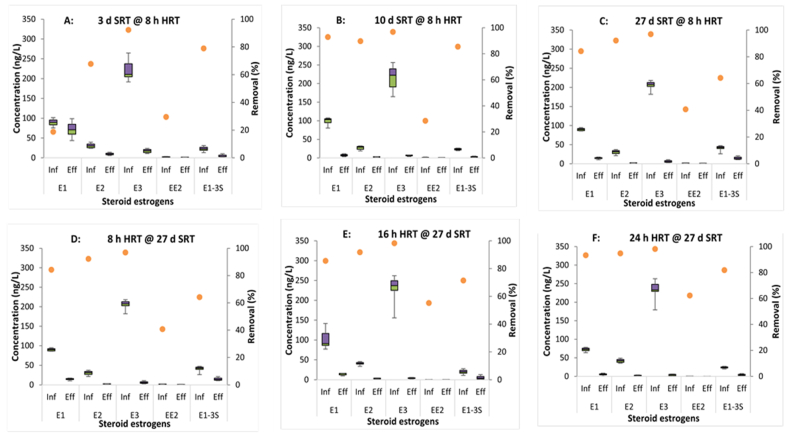
Boxplots of steroid estrogens concentrations in settled influent (Inf) and final effluent (Eff) in the pilot plant (n = 7); (A) 3 d, (B) 10 d and (C) 27 d SRT; (D) 8 h, (E) 16 h and (F) 24 h HRT. Percentage removal is indicated by the orange dots. Predicted no-effect concentration values for the estrogens are E1 = 3 ng/L; E2 = 1 ng/L; EE2 = 0.1 ng/L [41] and E3 = 60 ng/L [42].

Fig. 6 shows the influent and effluent concentrations of the estrogens as boxplots and their removals as scatterplots under different operational conditions. When operating at constant HRT, the removal extents for EE2 were 30, 29 and 41% at SRT of 3, 10 and 27 d respectively. Although the difference in removal at 3 and 10 d was negligible (*p* > 0.05, n = 7), a significant increase (12%) was observed from 10 to 27 d SRT (*p* < 0.05, n = 7). E1, E2, E3 and E1-3S were more effectively removed at 10 and 27 d SRT than at 3 d (Fig. 6). For constant SRT, the removal of all the estrogens showed progressive increases at 8, 16 and 24 h HRTs, ranging from 62 to 97% [40]. The removal of NP_6_EO and longer nonylphenol ethoxylates exceeded 85% and improved at 3, 10 and 27 d SRT (Table 2). However, shorter NPEOs (<NP_6_EO) showed significant improvement in removal as the SRT lengthened; with 44, 73 and 84% of NP_4_EO removed at 3, 10 and 27 d SRT respectively. At 3 d SRT, the concentration of short chain nonylphenols (mono-, di- and tricarboxylates) were higher in the effluent than settled sewage, but not at 10 and 27 d SRTs. This was due to limited degradation of short-chain NPEOs and the breakdown of long-chain NPEOs into short-chains at low SRT.

**Table 2:** Concentrations of nonylphenols in settled sewage and final effluent and their percentage removal from the pilot-scale study.

	Solids retention time (SRT)										Hydraulic retention time (HRT)								
	3 d				10 d			27 d			8 h			16 h			24 h		
	Settled sewage (ng/L)	Final Eff. (ng/L)	% Rem		Settled sewage (ng/L)	Final Eff. (ng/L)	% Rem	Settled Sewage (ng/L)	Final Eff. (ng/L)	% Rem	Settled sewage (ng/L)	Final Eff. (ng/L)	% Rem	Settled sewage (ng/L)	Final Eff. (ng/L)	% Rem	Settled sewage (ng/L)	Final Eff. (ng/L)	% Rem.
**NP**	1583	1738	**−10**	1062		1379	**−30**	1665	1764	**−6**	1665	1764	**−6**	247.9	73.2	**70**	628.9	234.0	**63**
**NP**_**1**_**EC**	48.8	207.1	**−324**	1374		443.4	**68**	45.3	101.5	**−124**	45.3	101.5	**−124**	629.7	2959	**−370**	416.2	1906	**−358**
**NP**_**2**_**EC**	68.0	1044	**−1435**	774.3		691.4	**11**	76.9	357.4	**−365**	76.9	357.4	**−365**	2334	3133	**−34**	1867	6108	**−227**
**NP**_**3**_**EC**	48.7	49.7	**−2**	48.8		681.0	**−1295**	45.3	94.6	**−109**	45.3	94.6	**−109**	155.1	832.5	**−437**	246.3	2117	**−759**
**NP**_**1**_**EO**	1812	710.7	**61**	65.7		1635	**−2391**	1067	372.8	**65**	1067	372.8	**65**	238.5	14.9	**94**	376.5	15.6	**96**
**NP**_**2**_**EO**	703.6	1066	**−52**	48.7		121.7	**−150**	1010	651.7	**35**	1010	651.7	**35**	51.4	3.5	**93**	153.2	3.4	**98**
**NP**_**3**_**EO**	10.4	18.0	**−73**	13.9		8.5	**39**	18.8	8.6	**54**	18.8	8.6	**54**	7.8	1.6	**80**	18.5	1.3	**93**
**NP**_**4**_**EO**	329.2	183.7	**44**	354.1		95.2	**73**	613.9	99.1	**84**	613.9	99.1	**84**	333.1	18.9	**94**	384.1	35.4	**91**
**NP**_**5**_**EO**	104.6	52.9	**49**	113.8		17.2	**85**	172.3	19.4	**89**	172.3	19.4	**89**	112.7	9.4	**92**	112.7	8.5	**92**
**NP**_**6**_**EO**	421.0	58.2	**86**	398.7		43.1	**89**	596.8	24.8	**96**	596.8	24.8	**96**	433.4	21.7	**95**	420.1	31.9	**92**
**NP**_**7**_**EO**	228.8	28.0	**88**	207.9		19.7	**91**	255.0	12.0	**95**	255.0	12.0	**95**	224.3	12.1	**95**	230.9	15.2	**93**
**NP**_**8**_**EO**	160.4	24.3	**85**	152.2		8.5	**94**	177.8	6.0	**97**	177.8	6.0	**97**	167.8	16.8	**90**	161.7	11.7	**93**
**NP**_**9**_**EO**	281.9	23.9	**92**	263.6		15.6	**94**	320.9	13.0	**96**	320.9	13.0	**96**	283.8	18.0	**94**	301.6	21.9	**93**
**NP**_**10**_**EO**	383.7	34.2	**91**	372.1		21.8	**94**	381.8	20.3	**95**	381.8	20.3	**95**	403.1	29.0	**93**	433.6	32.5	**93**
**NP**_**11**_**EO**	451.8	45.3	**90**	445.5		24.8	**94**	547.3	32.4	**94**	547.3	32.4	**94**	509.8	45.4	**91**	534.0	44.2	**92**
**NP**_**12**_**EO**	378.4	43.6	**88**	372.0		23.4	**94**	481.5	40.2	**92**	481.5	40.2	**92**	477.3	57.2	**88**	488.7	47.5	**90**

% Rem = % removal; Inf = influent; Eff = effluent.

### 3.7 Effect of SRT and HRT on estrogens and nonylphenols removal and microbial community structure

**Fig. 7:**
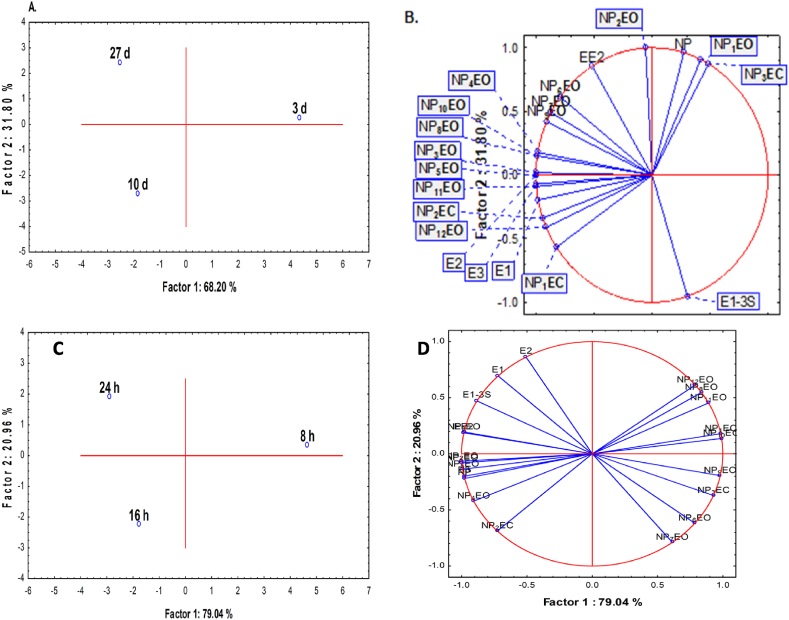
PCA and loading plots comparing the removal of nonylphenols and estrogens at 3, 10 and 27 d SRT (panels A and B) and at 8, 16 and 24 h HRT (panels C and D).

The removal of estrogens and nonylphenols rose as SRT and HRT increased, but between 10 and 27 d SRT the increase in removal was insignificant (Fig. 7, panel A). Nevertheless, Fig. 7, panel B indicates such difference can be due to deconjugation of E1-3S into E1 [43]. At constant SRT, estrogens and nonylphenols at HRTs showed high removals for the three HRTs (8, 16 and 24 h) (Fig. 7, panels C and D). The removals were significantly higher for estrogens and both short and long-chain nonylphenols, while the removal of only long chain nonylphenols were high (>84%) at 8 h HRT. However, the removal at 16 and 24 h HRTs are similar in the removal of estrogens and short-chain nonylphenols, while 8 h HRT stood apart due to its removal of the long-chain nonylphenols (>NP4EO). Breakdown of long-chain nonylphenols into short-chain is the reason why removal of short chains at low HRT is poor.

The dominance of *Proteobacteria* phylum observed from 16S rRNA analysis concurs with others [39,44]. At 3 d SRT, *Thiothrix eikelboomii*, the filamentous sulphur-oxidising bacterium responsible for bulking, loss of performance and sludge washout [45] dominated as also reported by Xie et al. [46]. The conditions in the ASP at 3 d SRT was ideal for *Thiothrix eikelboomii* because it is a heterotrophic bacterium capable of utilising a range of sugars and organic compounds as sole carbon sources; and a range of amino acids and ammonia as sole nitrogen sources [47,48]. Perhaps it was the high ammonia concentration in the mixed liquor that led to the dominance of *Thiothrix eikelboomii* at 3 d SRT [45]. At 10 d SRT, the *Thiothrix eikelboomii* abundance dropped by 85% and the ASP reactor was dominated by *Nitrospira* sp., leading to at 10.4 d SRT [49]. while, which may be due to the efficient removal of ammonia by the *Nitrospira species*. The decrease of *Thiothrix* sp. along with an increase in plant performance agrees with findings of Henriet et al. [45]. The ammonia-oxidising bacteria (AOB), *Nitrosomonas europaea* known to co-metabolise E2 and convert EE2 to nitro-EE2 when ammonia concentration is high, decreased as nitrification increased with SRT [50,51]. This may be due to increase in other AOBs which are complete ammonia oxidisers (comammox) and therefore an SRT increase at high HRT (F:M decrease) caused these slow-growing but highly energy-efficient comammox species to increase and maintain nitrification [52]. That is, increasing HRT reduces chemical loading rate and promotes competition among degraders [13] which changes the chemical degradation rate from first to zero order [53]. Therefore, a combination of long SRT and HRT enhances removal of estrogens and nonylphenols because it leads to enhancement in the mixing and contact between EDCs and the biomass, it changes the chemical removal rate from first-order to zero-order kinetics and it decreases the chemical loading rate.

## 4 Conclusion

This pilot-scale study has demonstrated that, activated sludge reactors have low microbial/bacterial diversity at low SRT which may contribute to its poor performance; and extending the SRT and HRT increased microbial/bacterial diversity in the reactor and improved the removal of estrogens and nonylphenols. *Thiothrix eikelboomii* was the dominant the bacteria species at 3 d SRT but diminished when the conditions in the bioreactor changed at 10 and 27 d SRT and the diversity increased. Removal of estrogens and nonylphenols generally increased with longer SRT and HRT, but short-chain nonylphenols concentrations in effluent was high at 3-d SRT due to the breaking down of long chain nonylphenols. Even at high SRT of 27 days and high HRT of 24 h, EE2 removal in activated sludge treatment process is low at 41%. The improved removal may be attributed to the increase in abundance and diversity of known and identified bacteria taxa capable of degrading estrogens and nonylphenols, although other removal routes such as sorption to sludge may have been enhanced.

## Author contribution statement

Lawson Mensah – Conceived and designed the experiments; Performed the experiments; Analyzed and interpreted the data; Wrote the paper.

Bruce Petrie – Contributed reagents, materials, analysis tools or data; Analyzed and interpreted the data.

Mark Scrimshawc – Conceived and designed the experiments; Analyzed and interpreted the data; Wrote the paper.

Elise Cartmell– Conceived and designed the experiments; Analyzed and interpreted the data.

Mandy Fletton– Contributed reagents, materials, analysis tools or data; Wrote the paper.

Pablo Campo - Conceived and designed the experiments; Analyzed and interpreted the data; Wrote the paper.

## Data availability statement

Data included in article/supp. material/referenced in article.

## Additional information

No additional information is available for this paper.

## Declaration of competing interest

The authors declare that they have no known competing financial interests or personal relationships that could have appeared to influence the work reported in this paper.
