# Supplementary material for: Influence of solids and hydraulic retention times on microbial diversity and removal of estrogens and nonylphenols in a pilot-scale activated sludge plant
Source: Heliyon. 2023 Aug 28;9(9):e19461. doi: 10.1016/j.heliyon.2023.e19461 (PMC10558614; doi:10.1016/j.heliyon.2023.e19461)
Supplement: Multimedia component 1 [file mmc1.pdf]

Supplementary Information

UPLC MS/MS detection

The *m/z* values for the detection of the estrogens and nonylphenols and operational parameters are presented by Petrie et al., (2013) and summarised as follows. For the estrogens, the parameters for the mass spectrometry detection were 3.20 kV capillary voltage, 650 V multiplier voltage, 1000 L/h desolvation gas flow, -55 V cone voltage, 0.2 V RF lens, 49 L/h cone gas flow; 350 °C desolvation temperature and 120 °C source temperature.

For the nonylphenols, the mass spectrometer was operated in the negative and positive electrospray ionisation mode utilising multiple reaction monitoring. The parameters for detection by the mass spectrometer were as follows: -2.3 kV (negative mode) and 3.20 kV (positive mode) capillary voltage; 1.0 V (negative mode) and 0.5 V (positive mode) RF lens, 3.0 V extractor lens, 650 V multiplier voltage, 1000 L/h desolvation gas flow, 50 L/h cone gas flow, 350 °C desolvation temperature and 120 °C source temperature (Petrie et al, 2014).

The limit of detection (LoD) and limit of quantification (LoQ) were derived based on the standard deviation of the response of blanks and the gradient. Seven blank samples were prepared by following the analytical procedure and analysed in the UPLC-MS/MS. The mean and standard deviation of the results were calculated using Excel 2016. Results were plotted and the concentration gradient was obtained. The LoD and LoQ were calculated as:

$$LoD = 3 \times SD / S$$ (**Equation 1**)

$$LoQ = 10 \times SD / S$$ (**Equation 2**)

where SD is standard deviation and S is the gradient.

UPLC-MS/MS method performance

The mean recoveries of the extraction of estrogens ranged between 85 and 101% for settled sewage and 89 – 98% for the final effluent. The nonylphenols recoveries were 55 – 98% and 58 – 99% for settled sewage and final effluent respectively. The relative standard deviations (RSD) of the recoveries were 6.4% or less in the estrogens. The limits of detections (LoD) of the estrogens were 0.04 – 0.17 ng/L in settled sewage and 0.03 – 0.13 ng/L in the final effluent. Estrone-1,3-sulfate had the lowest LoD in all samples and the least recovery in settled sewage. Recoveries of nonylphenol, nonylphenol carboxylates and nonylphenol ethoxylates in settled sewage ranged from 55 – 99% with RSD between 4.2 and 13.1%, whereas recoveries in final effluent were 58 – 99% with RSD from 1.6 to 12.8%. NP_4EO had poor recovery in all samples and high RSD. Long-chained ethoxylates had mostly poorer recoveries than short-chained ethoxylates. The LoDs of the NP, NPEOs and NPECs ranged from 1.5 to 47.9 ng/L in settled sewage and final effluent samples (Supplementary Table 1).

Supplementary Table 1. Limits of Quantification and Limits of Detection

Hazardous chemical	Settled sewage			Final effluent		
	LoD (ng/L)	*Recovery (%)*	*RSD (%)*	*LoD (ng/L)*	*Recovery (%)*	*RSD (%)*
**E1**	0.06	100.6	1.1	0.05	96.8	0.6
**E2**	0.17	92.1	6.4	0.13	96.7	6.8
**E3**	0.12	88.4	6.1	0.11	89.3	8.8
**EE2**	0.06	100.6	5.8	0.06	97.5	5.5
**E1-3S**	0.04	84.5	5.3	0.03	92.9	6.2
**NP**	23.8	99.2	4.2	9.3	98.9	3.3
**NP$_1$EC**	47.9	56.4	10.2	16.7	79.9	4.9
**NP$_1$EO**	30.7	97.5	4..4	14.2	84.0	6.3
**NP$_2$EO**	12.2	65.9	9.7	3.5	94.0	1.6
**NP$_3$EO**	3.7	61.5	13.1	1.5	62.2	5.5
**NP$_4$EO**	25.9	55.2	8.7	9.6	57.6	12.8
**NP$_5$EO**	11.1	58.5	11.8	2.4	75.8	8.1
**NP$_6$EO**	14.2	58.2	6.5	3.6	72.8	4.7
**NP$_7$EO**	8.0	68.4	5.2	3.1	75.2	3.9
**NP$_8$EO**	5.7	65.9	8.9	1.7	84.4	4.6
**NP$_9$EO**	9.1	62.5	5.5	2.8	75.9	3.3
**NP$_{10}$EO**	8.2	71.3	7.2	3.3	77.6	2.0
**NP$_{11}$EO**	11.4	55.9	12.8	4.1	63.8	7.9
**NP$_{12}$EO**	32.7	57.2	7.4	10.4	65.4	5.8

Limit of detection (LoD); relative standard deviation (RSD)

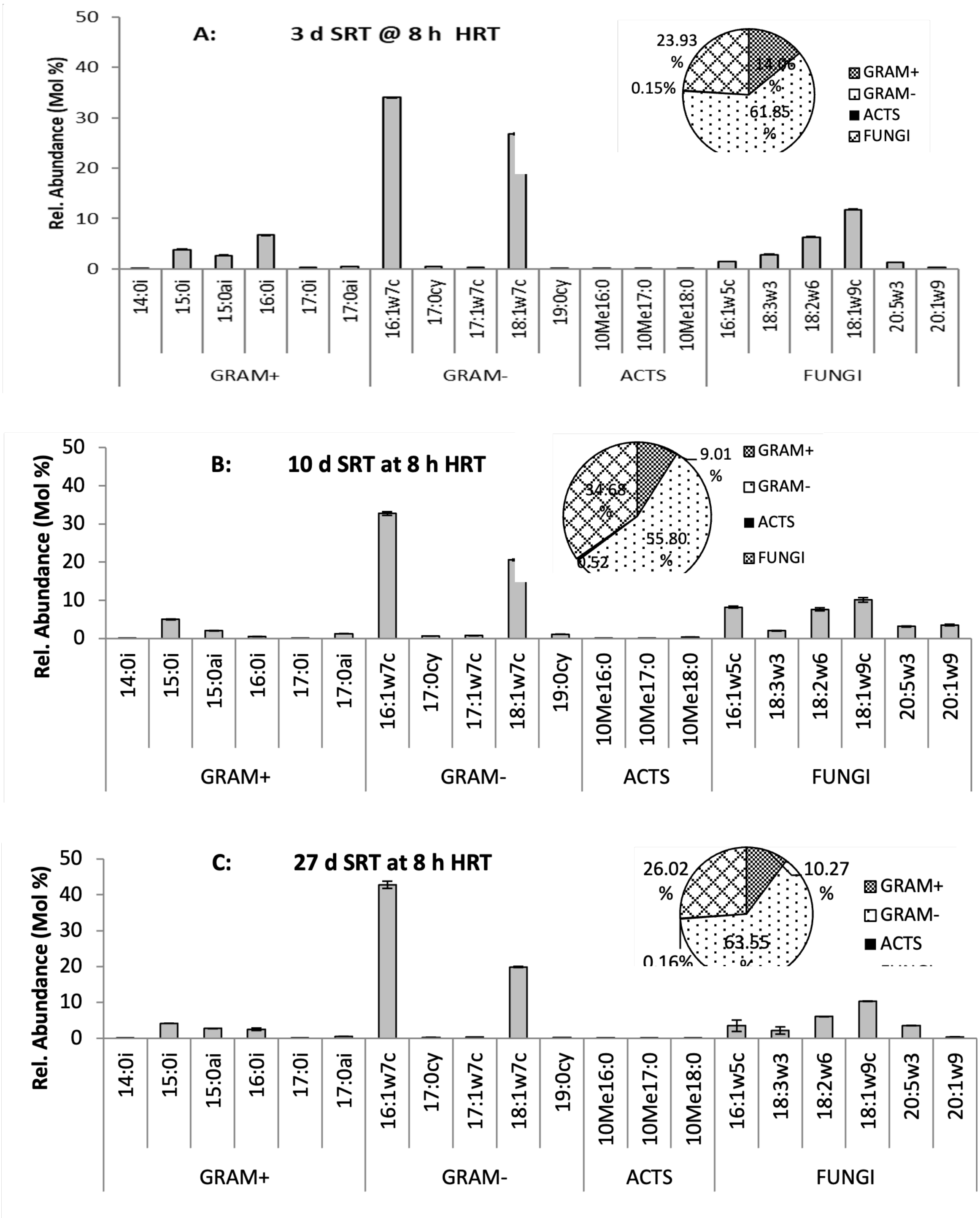

Supplementary Figure 1. Microbial diversity indicated by fatty acid methyl ester biomarkers at 3, 10 and 27 d SRT.

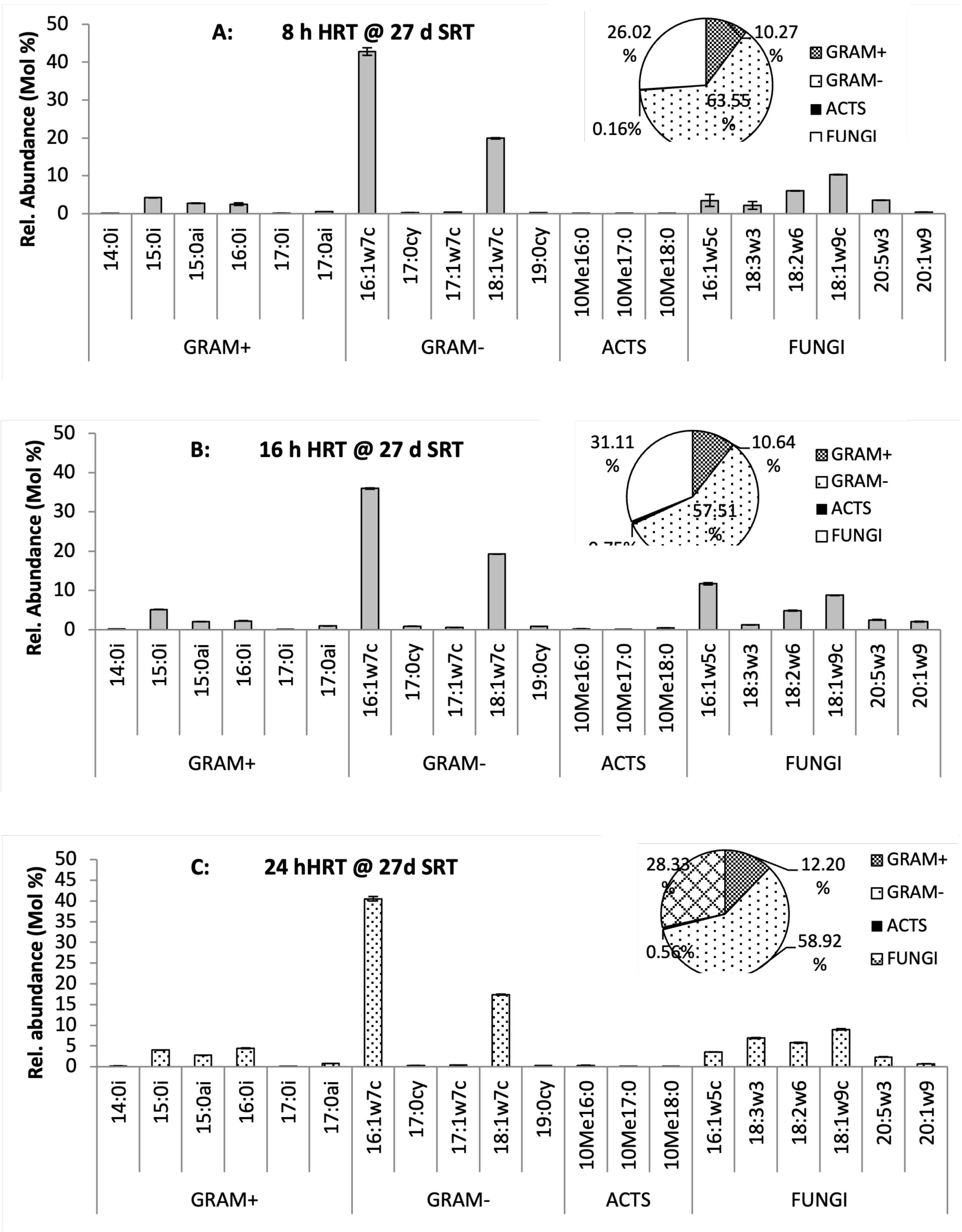

Supplementary Figure 2. Microbial diversity indicated by fatty acid methyl ester biomarkers at 8, 16 and 24 h HRT.

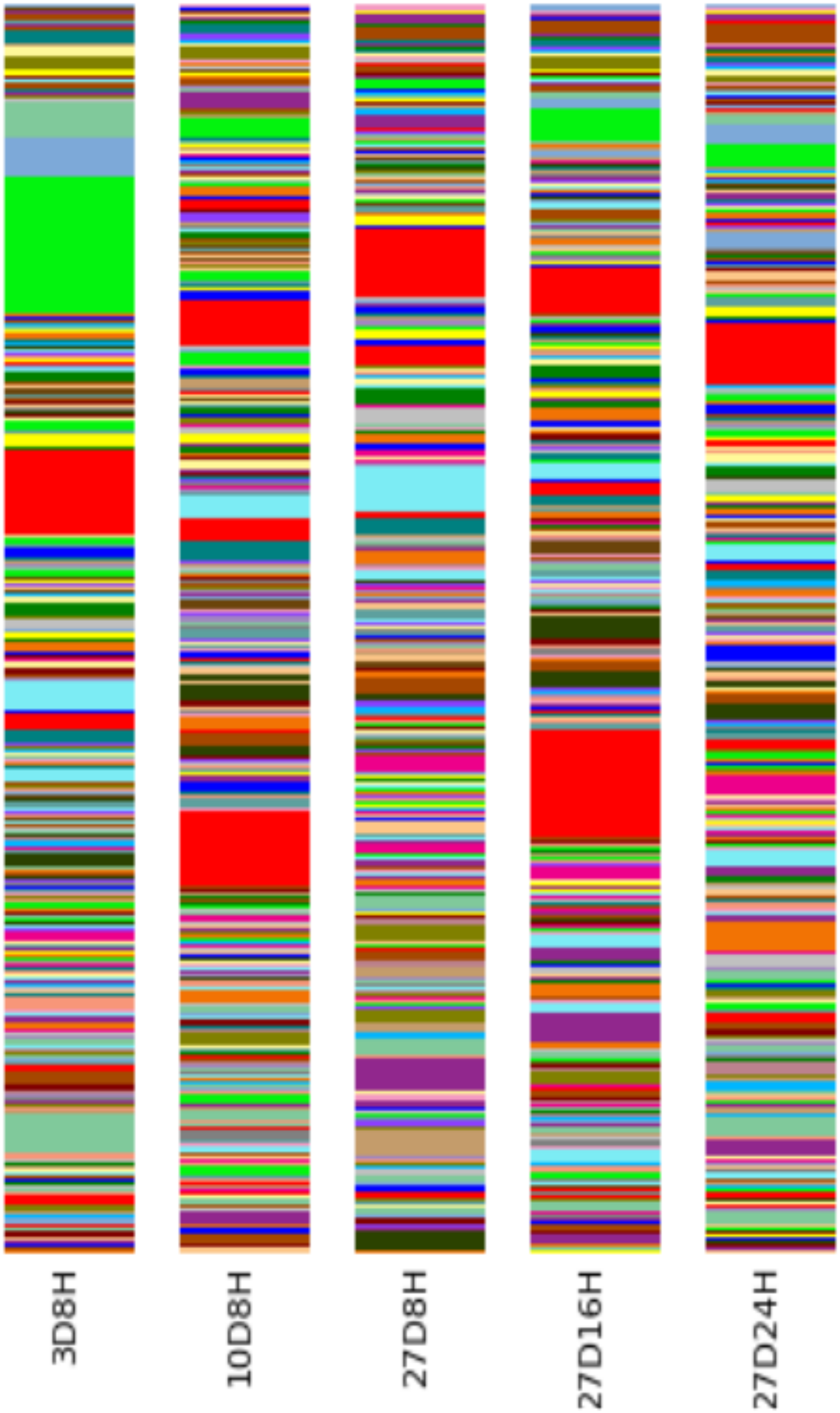

Supplementary Figure 3. Histogram of the bacterial species diversity at 3 d, 10 d and 27 d SRT and 8 h, 16 h and 24 h HRT.
